# Burned out Takayasu arteritis in a young caucasian female: A rare case of aortic regurgitation and aneurysm requiring surgical repair

**DOI:** 10.21542/gcsp.2025.38

**Published:** 2025-08-30

**Authors:** Zinya H Talukder, Enad Haddad, Asha Marwaha, Ross G. Biggs

**Affiliations:** 1 Jefferson Abington Hospital, Abington, PA; 2 Lehigh University, Bethlehem, PA; 3 Penn Medicine Lancaster General Hospital, Lancaster, PA

## Abstract

Takayasu arteritis (TAK), also known as pulseless disease, is a granulomatous vasculitis that affects the aorta and its primary branches. It predominantly affects Asian women aged 10 to 40 years and leads to occlusion, stenosis, and aneurysmal changes in large vessels. Aneurysmal changes are observed in the burned-out stage of TAK and are typically found in older patients. Here, we present a rare case of burned-out Takayasu arteritis in a 27-year-old Caucasian female presenting with isolated moderate-to-severe aortic regurgitation and aortic aneurysm requiring surgical repair. TAK initially presents with nonspecific symptoms; thus, diagnosis can be missed or delayed until late in the disease course. We highlight the need for high clinical suspicion, as delay in diagnosis and appropriate treatment can lead to significant morbidity and mortality.

## Introduction

Takayasu arteritis (TAK) is a rare granulomatous vasculitis characterized by damage to the large and medium-sized arteries and their branches. It has a reported worldwide incidence of 1 to 2 cases per million population. It demonstrates a female predominance, with a female-to-male ratio of 9:1. The average age of onset is 40–50 years. It is most commonly found in individuals of Asian or Mexican descent and is rare in North America^1^.

## Objective

We report a unique case of Takayasu arteritis in a 27-year-old Caucasian female presenting with isolated moderate-to-severe aortic regurgitation and aortic aneurysm requiring surgical repair. Furthermore, we provide a comprehensive review of the epidemiology, pathophysiology, risk factors, diagnostic methods, and management strategies for Takayasu arteritis.

## Case report

A 27-year-old Caucasian female with no significant rheumatologic or cardiac history presented with palpitations, headaches, vision changes, and a pounding sensation in her neck for 7 months. The patient reported no aggravating or alleviating factors. She denied any associated chest pain, shortness of breath, lightheadedness, dizziness, or diaphoresis. The patient’s vital signs were significant for blood pressure of 116/71 mmHg, heart rate of 98 beats/min, respiratory rate of 12 breaths/min, and oxygen saturation of 96% on room air.

**Figure 1.**
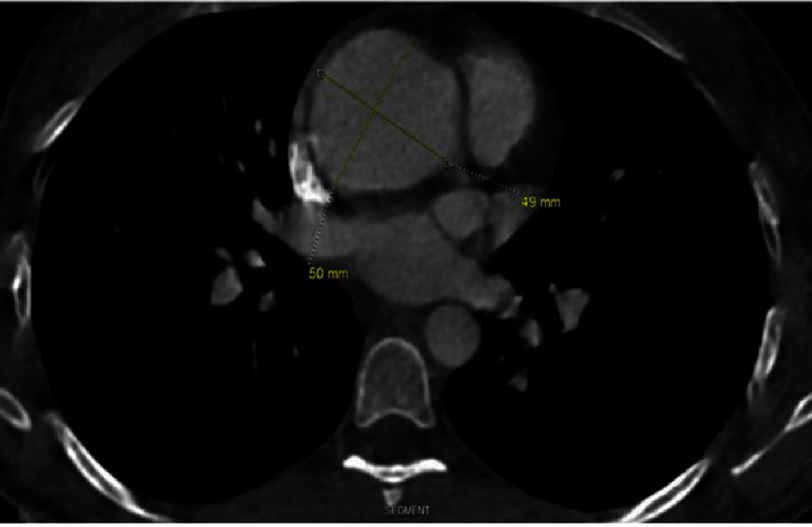
CT angiography of the chest demonstrating a 5.0 cm proximal aortic aneurysm.

On physical examination, the patient’s neurological and ophthalmologic examinations were unremarkable, and there were no signs of joint swelling, cyanosis, pulse differences between upper and lower extremities, or bruits. The patient’s pertinent laboratory workup is displayed in Table 1. An echocardiogram demonstrated an ejection fraction of 59% and a 4.9-cm aortic aneurysm with moderate-to-severe aortic insufficiency. CT angiography (CTA) of the chest showed a 5.0-cm proximal aortic aneurysm [Figure 1]. The patient underwent valve-sparing aortic root and ascending aortic replacement (David-V-Smod procedure) with a 24-mm Terumo Gelweave Valsalva graft.

**Table 1:** Table showing the patient’s laboratory values for various inflammatory markers and autoimmune workup.

	Patient’s Laboratory Value	Normal Range
C-Reactive Protein (CRP)	1.1 mg/L	0.00–5.00 mg/L
Erythrocyte Sedimentation Rate (ESR)	2.0 mm/hr	0–15 mm/hr
ANA	Negative	Negative
Anti-dsDNA	10 IU/mL	0–20 IU/mL
C3	90 mg/dL	88–201 mg/dL
C4	20 mg/dL	15–45 mg/dL

**Figure 2.**
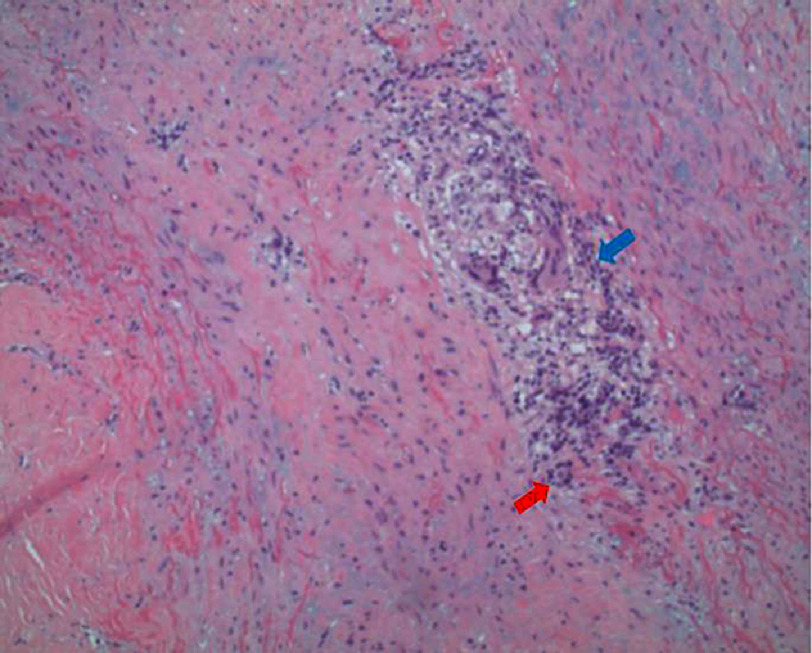
The patient’s aortic surgical pathology showing chronic inflammatory infiltrates (shown by blue arrow) with multinucleated giant cell infiltration (shown by red arrow).

Our patient experienced characteristic symptoms such as palpitations, headaches, and vision changes for more than 1 month, had moderate-to-severe aortic regurgitation and pulmonary artery lesions, thus meeting the modified Ishikawa criteria suggesting high probability of Takayasu arteritis [Table 2]. In addition, surgical pathology demonstrated chronic aortitis with giant cell infiltration consistent with Takayasu arteritis, thus confirming the diagnosis [Figure 2].

**Table 2:** Outline of the Major and Minor criteria of the Modified Ishikawa criteria for diagnosis of Takayasu arteritis met by our patient. The presence of two major, or one major and two minor, or four minor criteria suggests a high probability of Takayasu arteritis^1^. Specifically, our patient met one major and two minor criteria.

Major Criteria	Minor Criteria
Characteristic signs and symptoms, such as vision changes and palpitations, of at least 1 month duration	Aortic regurgitation or annulo-aortic ectasia
	Pulmonary Artery lesion

**Figure 3.**
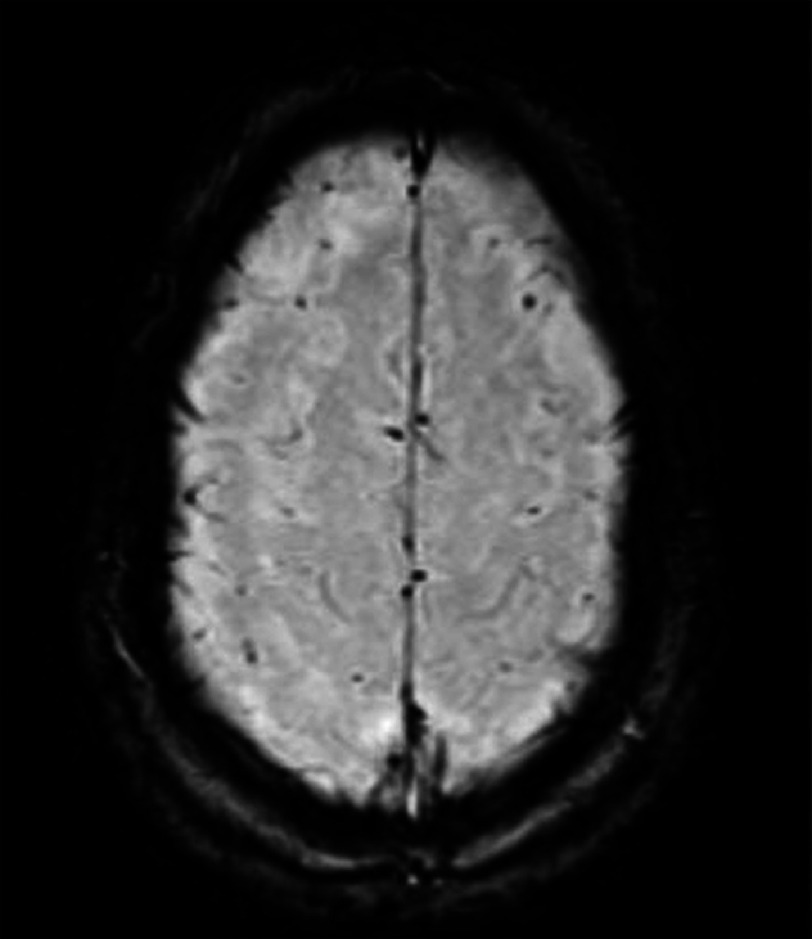
MRI of the brain demonstrating diffuse hemosiderin deposits reflecting remote hemorrhagic episodes, most likely secondary to arteritis. There is no evidence of intracranial hemorrhage, extra-axial collection, mass effect, or midline shift.

**Figure 4.**
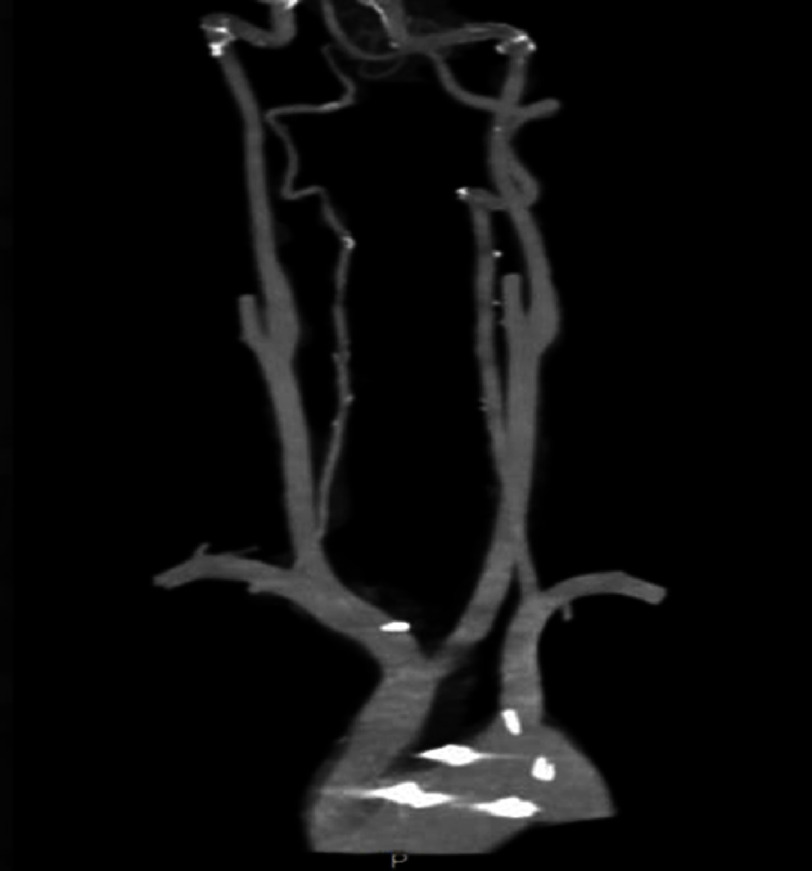
CTA of head and neck, demonstrating patent carotid and vertebral arteries, without any vascular malformation, thickening, aneurysm or stenosis.

On further imaging, MRI of the brain showed multiple hemosiderin deposits reflecting remote hemorrhagic episodes, most likely secondary to arteritis. It showed no evidence of recent hemorrhage or acute stroke [Figure 3]. CTA of the head and neck demonstrated tissue thickening of the walls of the aortic arch and proximal great vessels without significant stenosis. There was no significant carotid or vertebral artery thickening/ stenosis or evidence of CNS vasculitis [Figure 4].

**Figure 5.**
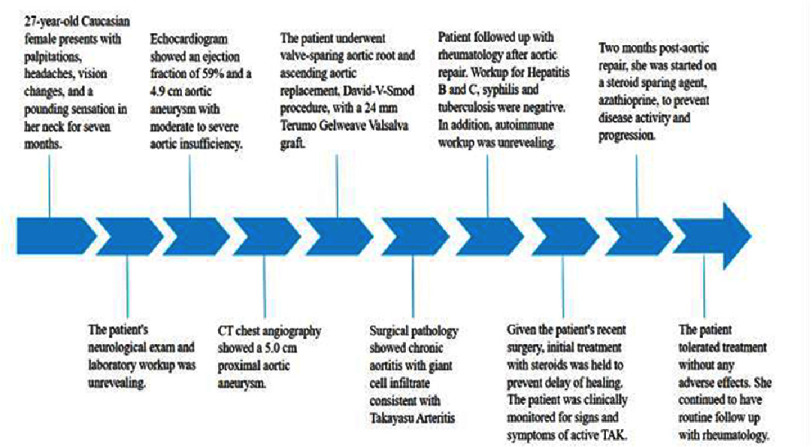
Visual summary of the patient’s clinical course.

The patient followed up with rheumatology after aortic repair. On further workup, other causes of vasculitis such as hepatitis B and C, syphilis, and tuberculosis were ruled out [Table 3]. In addition, autoimmune workup including ANA, anti-dsDNA, C3, and C4 was unrevealing [Table 1]. Given the patient’s recent surgery, initial treatment with steroids was withheld to prevent delayed healing. Instead, the patient was clinically monitored for signs and symptoms of active TAK. Two months post-aortic repair, she was started on a steroid-sparing agent, azathioprine, to prevent disease activity and progression. She tolerated treatment well without any adverse effects. See Figure 5 for a timeline of these events.

**Table 3:** Infectious disease workup performed to rule out other causes of vasculitis.

	Patient’s Laboratory Value	Normal Range
Hepatitis B Surface Antigen (HBsAg Screen)	Negative	Negative
Hepatitis C Antibody	Nonreactive	Nonreactive
Syphilis (Treponema Pallidum Antibodies)	Nonreactive	Nonreactive
Tuberculosis (QuantiFERON-TB Gold Plus)	Negative	Negative

## Discussion

Our 27-year-old Caucasian patient was found to have isolated moderate-to-severe aortic regurgitation and aortic aneurysm requiring surgical repair represents a unique case of TAK.

Although Takayasu arteritis progresses through three stages, this might represent an oversimplification of a complex disease process. In the early systemic stage (pre-vasculitis stage), patients generally experience constitutional symptoms such as fatigue and low-grade fevers. In the second stage (vascular inflammatory stage), patients may experience arthralgia, neurological symptoms such as headache or syncope, vascular pain such as carotidynia, and symptoms of vascular insufficiency such as limb claudication or vision changes. Vascular stenosis and aneurysmal changes occur during the vascular inflammatory stage. In the third stage (burned-out stage), fibrosis occurs, patients are considered to be in remission, and they experience minimal symptoms^3^. Our patient presented with several months of neurological symptoms, specifically headaches and a pounding sensation in her neck for 7 months. She denied experiencing any constitutional symptoms prior to her presentation. Although her symptoms were suggestive of the second stage of TAK, her inflammatory markers were within normal limits [Table 1], and her neurological examination was unremarkable, suggesting she had progressed into the third stage of TAK.

It is important to note that although inflammatory markers such as C-reactive protein (CRP) and erythrocyte sedimentation rate (ESR) are commonly used as indicators of inflammation and disease activity, there is evidence suggesting that these markers are not reliable for distinguishing active from inactive TAK. ESR has a sensitivity and specificity for active TAK of 72% and 56%, respectively. CRP has sensitivity and specificity of 71.4% and 100%, respectively. In contrast, imaging modalities such as magnetic resonance angiography (MRA) have sensitivity and specificity of 100%, and CTA has sensitivity and specificity of 95% and 100%, respectively^4^. One case-control study demonstrated that high-sensitivity CRP was a significant predictor of major cardiac events^5^. In addition, patients with active disease tend to have higher levels of circulating interleukin (IL)-6 and IL-18^5^. Unfortunately, circulating IL-6 and IL-18 were not evaluated in our patient.

In TAK, stenotic and occlusive changes result in arterial insufficiency and lead to symptoms of limb claudication and vision changes. Arterial dilation and aneurysm occur in 2.8–31.9% of patients. There are limited reports of TAK with isolated aneurysms in the absence of occlusive lesions^6^. Our patient’s echocardiogram showed an ejection fraction of 59% and a 4.9-cm aortic aneurysm with moderate-to-severe aortic insufficiency. CT angiography (CTA) of the chest showed a 5.0-cm proximal aortic aneurysm [Figure 1]. The patient’s CTA of the head and neck did reveal tissue thickening of the walls of the aortic arch and proximal great vessels. However, there was no significant stenosis. Aneurysmal changes are reported mainly in patients older than 40 years, while our patient was 27 years old^7^.

The pathophysiology of TAK involves a complex interaction between immune-mediated processes, vascular remodeling, and genetic factors. The early stage involves active inflammation, necrosis, and infiltration of mononuclear cells. Chronic inflammation leads to thickening of the arterial wall, resulting in stenosis and occlusion. In rapid or severe inflammation, the destruction of smooth muscle cells results in weakening of the arterial wall, leading to vascular dilatation and aneurysm formation. The burned-out phase of TAK involves adventitial fibrosis and scarring, along with persistent lymphoplasmacytic inflammation and multinucleated giant cells^8^. The surgical pathology report for our patient, which showed chronic aortitis with multinucleated giant cell infiltration, is suggestive of the burned-out phase of TAK [Figure 2].

Takayasu arteritis shares many histologic and clinical features with giant cell (temporal) arteritis and other large-vessel vasculitides. Therefore, differential diagnosis for our patient included giant cell (temporal) arteritis and other large-vessel vasculitides such as Cogan syndrome and Behçet syndrome. Giant cell arteritis also affects the aorta; it has a predilection for the cranial branches of the aortic arch. However, our patient’s CTA of the head and neck revealed tissue thickening of the walls of the aortic arch and proximal great vessels. In addition, the incidence of GCA increases with age and peaks between 70 and 80 years of age^5^. In contrast, TAK is considered a disease of the young, with most patients between 40 and 50 years of age^2^. As a result, GCA was thought to be a less likely cause of the patient’s symptoms. Therefore, temporal artery biopsy was not considered. Cogan syndrome is a chronic inflammatory disorder that most commonly affects young adults between 5 and 63 years of age. However, clinical hallmarks are interstitial keratitis, referring to inflammation of the cornea causing eye redness and pain, and vestibuloauditory dysfunction such as hearing loss and ataxia, which were not seen in our patient^8^. Behçet syndrome can involve dilatation and aneurysms of medium- and large-sized arteries. However, patients usually have oral or genital ulcerations, venous thrombosis, ocular disease, and arthritis, which were not seen in our patient^5^.

CTA is the standard for initial staging of disease distribution in TAK. The mainstay of therapy includes immunosuppressive agents and systemic glucocorticoids. Treatment options are determined by the patient’s disease severity. Angiography is used to classify patients with TAK. Specifically, mild disease refers to patients who do not have evidence of new arterial stenosis, aortitis, carotidynia, or critical ischemia. Moderate disease refers to patients with new or progressive arterial lesions that are mild. In contrast, severe disease refers to patients with arterial stenosis resulting in symptoms. Mild active disease is usually treated with a combination of adalimumab (tumor necrosis factor inhibitor) and moderate-dose glucocorticoid. Methotrexate or azathioprine in combination with moderate-dose glucocorticoid are alternative treatment options for patients who are unable to use TNF inhibitors due to cost, contraindications, or patient/clinical preference.

Patients with moderate or severe active disease are treated with a combination of adalimumab (tumor necrosis factor inhibitor) and high-dose glucocorticoid. For patients who are unable to use TNF inhibitors, tocilizumab, an interleukin-6 (IL-6) inhibitor, is recommended^5^. Thus far, there are no randomized trials examining the use of glucocorticoid monotherapy for TAK treatment. However, there are some observational data suggesting that glucocorticoid monotherapy may be adequate to achieve remission in up to half of patients. However, over half of patients treated with glucocorticoid alone have been shown to relapse and require further treatment with steroids, leading to toxicity from extended and repeated courses of glucocorticoids^5^. Therefore, the currently recommended treatment is steroid-sparing agents in combination with glucocorticoids. A recent nationwide study showed that 50% of patients with Takayasu arteritis will experience relapsing disease and vascular complications within 10 years of diagnosis. In addition, male patients and those with elevated C-reactive protein were more likely to experience relapsing disease^2^.

Although traditionally surgical therapy has been reserved for symptomatic manifestations of arterial occlusive disease refractory to medical therapy, surgical revascularization is evolving as a primary treatment option^5^. Vascular intervention may be necessary for the treatment of stenosed, occluded, or aneurysmal disease. Progressive aneurysmal dilation can result in dissection, rupture, or severe aortic regurgitation^9^. Our patient’s echocardiogram showed a 4.9-cm aortic aneurysm with moderate-to-severe aortic insufficiency. CT angiography of the chest confirmed the proximal aortic aneurysm. She required surgical repair; specifically, she underwent valve-sparing aortic root and ascending aortic replacement. Given the patient’s recent surgery, initial treatment with steroids was withheld to prevent delayed healing. The patient was instead closely monitored for signs and symptoms of active TAK. Two months after aortic repair, the patient was started on a steroid-sparing agent, specifically azathioprine, as TNF inhibitors were too expensive for the patient, to prevent disease activity and progression.

Routinely, patients are tested for thiopurine methyltransferase enzyme activity by enzyme activity or genetic testing prior to initiating azathioprine^5^. However, this was not performed on our patient; instead, her complete blood count was monitored as the azathioprine dose was uptitrated. Typically, azathioprine can be initiated at 50 mg/day with an increase every 1 to 2 weeks, if the patient’s white blood cell count remains stable, until the goal dose is obtained^5^. Our patient underwent close follow-up with rheumatology approximately every 6 months. The patient tolerated her treatment well without any adverse reactions, which are commonly seen with azathioprine therapy, such as diarrhea, nausea/vomiting, symptoms of myelosuppression, and infections. The patient’s ESR and CRP were monitored every 6 months, and these laboratory values remained negative. In addition, 1 year after diagnosis, she underwent repeat CT chest imaging, which did not show any new aneurysmal or stenotic changes.

The decision to discontinue treatment is complex due to the highly variable course of TAK as well as the extended time between disease exacerbations. Immunosuppressive therapies should be tapered only after detailed discussion with the patient regarding the risk of chronic immunosuppression and the risk of progressive arterial damage. It is recommended that patients have at least 2 years of inactive disease before considering tapering treatment. In addition, positron emission tomography (PET) scans are often used to help with the decision to reduce immunosuppressive therapy^5^. However, this was not obtained for our patient. The patient has been maintained on azathioprine monotherapy, and she has not experienced any recurrence of her neurological symptoms. In addition, the patient’s immunosuppressive therapy has not been tapered yet.

## Conclusion

Diagnosis of TAK can be challenging as patients initially present with nonspecific symptoms. However, missed or delayed diagnosis can lead to significant morbidity and mortality. Our atypical case of TAK in a 27-year-old Caucasian female with significant aortic insufficiency and aortic aneurysm requiring surgical repair, who otherwise had no signs or symptoms of active TAK, highlights the need for high clinical suspicion for this disease.

### What have we learned?

∘ Takayasu arteritis (TAK) is a rare granulomatous vasculitis characterized by damage to the large and medium-sized arteries and their branches, with female predominance, mainly affecting women between 10 and 40 years of age.
∘ TAK initially presents with nonspecific symptoms; therefore, diagnosis can be missed or delayed until late in the course of the disease.
∘ CTA is the standard for initial staging of disease distribution in TAK.
∘ The mainstay of therapy includes a combination of steroid-sparing agents such as adalimumab, methotrexate, or azathioprine and glucocorticoids.
∘ The decision to discontinue treatment is complex due to the highly variable course of TAK and the extended time between disease exacerbations. Shared decision-making and routine laboratory workup are paramount to monitor disease relapse.

## Limitations

Our patient did not undergo a complete autoimmune workup. For example, antineutrophil cytoplasmic antibodies (ANCA), anti-Ro antibodies, anticardiolipin antibodies, circulating immune complexes, and anti-endothelial cell antibodies (AECA) were not performed. Although initially glucocorticoids were withheld to prevent delayed healing after the patient’s surgical procedure, there was no documentation as to why glucocorticoids were not considered later, as a combination of steroid-sparing agents and glucocorticoids is standard therapy. In addition, although our patient underwent repeat CT chest imaging after 1 year, positron emission tomography (PET) scan was not obtained to determine whether to reduce immunosuppressive therapy.

Our case report did not require IRB approval.
